# Risk Analysis Index as a Predictor of Mortality and Failure to Rescue in Patients Undergoing Distal Femur Fracture Fixation

**DOI:** 10.1007/s43465-025-01610-3

**Published:** 2025-11-08

**Authors:** Kush Modi, Omar Sbaih, Jared Sasaki, Victor Koltenyuk, Nithin Gupta, Hursch Patel, James D. Miller

**Affiliations:** 1 Georgetown University School of Medicine, Washington, DC USA; 2 Bowers Neurosurgical Frailty and Outcomes Data Science Lab, Flint, MI USA; 3 School of Medicine, New York Medical College, 40 Sunshine Cottage Road, Valhalla, NY 10595 USA; 4 Department of Orthopaedic Surgery, Jefferson Health NJ, Stratford, NJ USA; 5 Department of Orthopaedic Surgery, OhioHealth Doctors Hospital, Columbus, OH USA

**Keywords:** Distal femur fractures, Frailty, Risk Analysis Index, Failure to rescue, Mortality

## Abstract

**Purpose:**

This study sought to evaluate the use of frailty, compared to age alone and American Society of Anesthesiologists (ASA) score, for prediction of mortality and failure to rescue (FTR) in patients undergoing surgical fixation of distal femur fractures (DFFs).

**Methods:**

The American College of Surgeons National Surgical Quality Improvement Program (ACS-NSQIP) database was queried for patients who underwent surgical management of DFFs. Frailty was evaluated using the 5-Item Modified Frailty Index (mFI-5) and Risk Analysis Index (RAI). Outcomes included 30-day mortality and FTR (mortality following major complication). Multivariate regression and receiver operating characteristic curve (AUROC) analyses were performed to assess odds ratio (OR) and discriminatory accuracy (quantified by C-statistic), respectively.

**Results:**

There were 2638 patients (median age 73 years) undergoing DFF fixation included in this study. Of these, 90 (3.4%) patients experienced mortality, while FTR occurred in 51 (1.9%) patients. Increasing frailty, as rated by the mFI-5 and RAI, was significantly associated with higher odds of 30-day mortality and FTR in patients undergoing surgical fixation of DFF. Additionally, RAI showed superior discriminatory accuracy for 30-day mortality and FTR compared to mFI-5, age, and ASA.

**Conclusions:**

The RAI demonstrates superior predictive value and discriminatory accuracy for postoperative mortality. Given these findings, frailty as measured by the RAI may be a useful bedside screening tool to identify patients at risk for adverse outcomes. In doing so, patients can be optimized preoperatively to improve clinical care.

## Introduction

Distal femur fractures (DFF) are associated with high rates of morbidity and mortality and are common in the elderly population following low-energy trauma [1]. Previous literature suggests the 30-day mortality rate is between 6 to 10%, with the 1 year mortality rate ranging higher from 9 to 25% [2, 3]. The high mortality rates among DFF patients may be attributed to the fact that these fractures are more common among elderly individuals [4]. DFFs also present a major clinical challenge because they are often technically difficult to fix, and a substantial proportion of patients are poor surgical candidates due to advanced age, frailty, or multiple comorbidities [5]. With the aging population in the United States, the prevalence of DFFs is expected to rise, highlighting the need to understand their impact on elderly individuals. Preoperative risk stratification tools, such as frailty, are becoming increasingly utilized to aid in surgical decision-making and identify patients at greater risk for adverse postoperative outcomes.

Within the field of orthopedics, one of the most commonly used scoring systems to assess frailty is the 5-Item Modified Frailty Index (mFI-5) [6]. The mFI-5 score is based on the presence of five comorbidities, including congestive heart failure within 30 days prior to surgery, insulin- or noninsulin-dependent diabetes mellitus, chronic obstructive pulmonary disease or pneumonia, partially or totally dependent functional health status at time of surgery, and hypertension requiring medication [7]. In comparison, the more recently developed Risk Analysis Index (RAI) offers a broader assessment of frailty by incorporating functional status, social support, cognitive ability, and nutritional health alongside comorbidities [8]. This comprehensive approach allows the RAI to more accurately capture frailty, and it has demonstrated stronger predictive performance and discriminatory ability than the mFI-5 across neurosurgical contexts, including various spine surgeries [9, 10]. The RAI has been externally validated and has already started to become incorporated into electronic medical records (EMRs) [11].

Currently, the American Society of Anesthesiologists (ASA) score is frequently used to determine a patient’s surgical risk preoperatively [12]. However, when comparing ASA scores to frailty measures, it is unclear which one has superior predictive value and discriminatory accuracy for postoperative mortality. Recently, frailty has emerged as a tool of risk stratification preoperatively, but its specific benefits related to orthopedic trauma remains limited. Particularly, there are no studies that compare RAI to other risk stratification tools for DFF fixation patients. Therefore, the purpose of this study is to investigate the use of RAI vs. other measures in predicting mortality and failure to rescue (FTR) rates in patients undergoing surgical fixation of DFFs.

## Materials and Methods

### Data Source

Data was collected from the American College of Surgeons National Surgical Quality Improvement Program (ACS NSQIP) database. The ACS NSQIP is a nationally validated, multi-institutional database that includes risk-adjusted preoperative information and 30-day postoperative outcomes. No Institutional Review Board (IRB) review was necessary for this study.

### Patient Selection Criteria

Patients included in this study were identified by using the *Current Procedural Terminology* (CPT) codes 27,511 and 27,513 for open treatment of femoral supracondylar or transcondylar fracture without or with intercondylar extension, respectively. CPT code 27,514 was also used for identifying patients that underwent open treatment of DFF involving the medial or lateral condyle. The 10th revision of the *International Classification of Diseases* (ICD 10) codes were applied for DFFs (S72.401, S72.402, S72.409), and displaced femoral condyle fractures (S72.411, S72.412). Patients with polytrauma, periprosthetic fractures, or pathologic fractures were excluded.

### Frailty Measurements

Frailty was evaluated using the modified 5-item frailty index (mFI-5) and the risk analysis index (RAI). The mFI-5 measures frailty by assigning one point for each of the five functional status and comorbidity variables present in a patient. These variables include diabetes, hypertension, congestive heart failure (CHF), chronic obstructive pulmonary disease (COPD), and functional dependence. The cutoffs used to categorize patients to different frailty status based on mFI-5 scores were as follows: normal (mFI-5 = 0 or 1), frail (mFI-5 = 2), and very frail (mFI-5 ≥ 3). RAI is a more comprehensive measure of frailty, accounting for 11 weighted variables encompassing demographics, functional status, comorbidities, and laboratory values, and each variable was matched to the variables in the NSQIP database. ‘Age’ in RAI was matched to ‘age at surgery’ in NSQIP, ‘sex’ to ‘sex,’ ‘cancer’ to ‘disseminated cancer or chemotherapy within 30 days,’ ‘weight loss’ to ‘ > 10% body weight loss in 6 months,’ ‘functional dependence’ to ‘functional status prior to surgery,’ ‘renal failure’ to ‘renal failure requiring dialysis,’ ‘CHF’ to ‘congestive heart failure within 30 days,’ ‘dyspnea’ to ‘dyspnea at rest or on exertion,’ ‘COPD’ to ‘history of COPD,’ and ‘cognitive impairment’ to ‘impaired sensorium or coma.’ Patients were classified into frailty tiers using RAI scores as follows: normal (RAI < 30), frail (RAI 30–40), and very frail (RAI > 40).

### Demographic and Clinical Characteristics

Descriptive demographic variables that were collected for the total cohort included age, sex, and race. Preoperative variables were also collected, including ASA classification, functional status (independent, partially dependent, or totally dependent), and preoperative comorbidities within 30 days prior to surgery. Some of these comorbidities include hypertension, COPD, insulin-dependent and non-insulin dependent diabetes mellitus (DM), and bleeding disorders. Clinical intraoperative and postoperative variables were also collected for the total cohort. Operative characteristics were categorized as elective or nonelective, and inpatient or outpatient. Postoperative complications data, and discharge destination data, including rehabilitation facility, skilled nursing care facility, and home, were collected. Data was reported as total counts with percentages.

### *Primary and Secondary Outcomes*

The primary outcomes were 30-day mortality and FTR. FTR is a standardized measure created by the Agency for Healthcare Research and Quality (AHRQ) that was defined as death within 30 days after a major complication. Major complications included the following: pneumonia, reintubation, pulmonary embolism, and myocardial infarction. Minor complications included intra- or postoperative blood transfusion and urinary tract infection. Secondary outcomes included incidence of major complications and minor complications, unplanned 30-day readmission and 30-day reoperation, and non-home discharge (NHD).

### Statistical Analysis

The IBM SPSS Software was used to conduct the statistical analysis. Multivariate regression models were performed using full models with variables selected based on clinical relevance and prior literature. Variables controlled for included age, sex, race, ethnicity, inpatient status, operative time, and total length of stay to evaluate frailty as a predictor of the primary and secondary endpoints. Operative time and length of stay were included in the multivariate analysis in an attempt to control for confounding variables that may occur intraoperatively during DFF fixation procedures. Results of multivariate logistic regression are represented as odds ratios (OR) with 95% confidence intervals (95% CI). Receiver Operating Characteristic (ROC) curve analysis was performed to assess the discriminatory accuracy of RAI and mFI-5 models in predicting postoperative outcomes. The area under the ROC curve (AUROC), or C-statistic, was quantified using the DeLong test. Analysis of variance (ANOVA) was used for post hoc testing. Statistical significance was considered with a p-value of < 0.05. Due to the retrospective nature of the study and large sample size, no formal power analysis was performed.

## Results

### Cohort Characteristics

There were 2638 patients (females: 2096, 79.5%) with a median age of 73 years (IQR 53–93) who were included in the study cohort (Table 1). The most common preoperative comorbidities were hypertension (66.0%, N = 1,742), diabetes mellitus (DM) (27.8%, N = 734), and bleeding disorder (13.9%, N = 368).

**Table 1:** Demographics, clinical characteristics, discharge destinations, and post-operative outcomes and complications of patients undergoing distal femoral fracture fixation. (N = 2,638)

Variable	Value
Total Cohort (N)	2,638
Median Age (IQR), years	73 (53–93)
Sex (M/F)	542 (20.5%)/2096 (79.5%)
Race	
White	2,021 (76.6%)
Black	210 (8.0%)
Asian	43 (1.6%)
Other/Unknown	364 (13.8%)
Functional status	
Independent	2,155 (81.7%)
Partially dependent	391 (14.8%)
Totally dependent	92 (3.5%)
ASA classification	
1-No disturbance	51 (1.9%)
2-Mild disturbance	530 (20.1%)
3-Severe disturbance	1,630 (61.8%)
4-Life threatening	427 (16.2%)
RAI category	
Normal	2,146 (81.3%)
Frail	392 (14.9%)
Very frail	100 (3.8%)
mFI-5 category	
Normal	1,656 (62.8%)
Frail	740 (28.1%)
Very frail	242 (9.2%)
Comorbidities	
COPD	223 (8.5%)
Congestive heart failure (CHF)	89 (3.4%)
Hypertension	1,742 (66.0%)
Renal failure	36 (1.4%)
Dialysis	92 (3.5%)
Steroid use	131 (5.0%)
Bleeding disorder	368 (13.9%)
Insulin-dependent diabetes	430 (16.3%)
Non-insulin dependent diabetes	304 (11.5%)
Operation type	
Elective	429 (16.3%)
Nonelective	2,205 (83.6%)
Inpatient	2,561 (97.1%)
Outpatient	77 (2.9%)
Discharge destination	
Home	724 (27.4%)
Rehab	436 (16.5%)
Skilled care	1,254 (47.5%)
Postoperative complications	
Pneumonia	74 (2.8%)
Superficial Surgical Site Infection (SSI)	25 (0.9%)
Reintubation	37 (1.4%)
Pulmonary embolism	32 (1.2%)
Urinary tract infection	126 (4.8%)
Myocardial infarction	34 (1.3%)
Blood transfusion	863 (32.7%)
Postoperative outcomes	
Mortality	90 (3.4%)
Major complication	176 (6.7%)
Minor complication	989 (37.5%)
Readmission	198 (7.5%)
Reoperation	78 (3.0%)
Failure to rescue (FTR)	51 (1.9%)
Non-home discharge (NHD)	1,914 (72.6%)

*IQR* Interquartile range, *COPD* Chronic obstructive pulmonary disease, *ASA* American Society of Anesthesiologists, *RAI* Risk Analysis Index, *mFI-5* 5-Item Modified Frailty Index

Regarding frailty status, the mFI-5 score classified 28.1% (N = 740) of patients as frail and 9.2% (N = 242) of patients as very frail. In assessing frailty using RAI, 14.9% (N = 392) of patients were classified as frail and 3.8% (N = 100) were classified as very frail. Regarding ASA classification, approximately 78% of patients had a Class III or higher (N = 2,057).

In terms of postoperative complications, the most common were blood transfusion (32.7%, N = 863), urinary tract infection (4.8%, N = 126), and pneumonia (2.8%, N = 74). Regarding 30-day postoperative outcomes, a 3.4% (N = 90) mortality rate and a 1.9% (N = 51) failure to rescue (FTR) rate were reported. Most patients were discharged to skilled care facilities (47.5%, N = 1,254) followed by home (27.4%, N = 724) and rehabilitation facilities (16.5%, N = 436).

### Primary Outcomes

Multivariate regression was performed to assess the predictive value of RAI and mFI-5 on 30-day postoperative outcomes (Table 2). As assessed by RAI, classification of patients as frail had greater odds of mortality (OR = 2.11, 95% CI (1.24–3.58), p-value =  < 0.01) compared to normal patients. RAI classification of very frail was also predictive of greater risk of mortality (OR = 6.36, 95% CI (3.36–12.04) p-value =  < 0.01), and FTR (OR = 2.57, 95% CI (1.02–6.43), p-value =  < 0.05) compared to normal patients. Comparatively, very frail patients as characterized by mFI-5 had greater odds of mortality (OR = 3.61, 95% CI (2.07–6.27), p-value =  < 0.01), and FTR (OR = 3.48, 95% CI (1.74–6.97), p-value =  < 0.01) compared to normal patients.

**Table 2:** Results of the multivariate regression analysis controlling for age, sex, race, ethnicity, inpatient status, operative time, and total length of stay for the prediction of 30-day outcomes by frailty as measured by the RAI or mFI-5

	RAI		mFI-5	
	Frail (OR, 95% CI)	Very frail (OR, 95% CI)	Frail (OR, 95% CI)	Very frail (OR, 95% CI)
Mortality	2.11 (1.24–3.58)**	6.36 (3.36–12.04)**	1.25 (0.75–2.07)	3.61 (2.07–6.27)**
Failure to rescue	1.67 (0.85–3.28)	2.57 (1.02–6.43)*	1.04 (0.52–2.07)	3.48 (1.74–6.97)**
Major complication	1.45 (0.97–2.18)	2.40 (1.32–4.38)**	1.54 (1.09–2.19*	2.99 (1.94–4.62)**
Minor complication	1.25 (0.99–1.58)	1.47 (0.96–2.23)	1.28 (1.07–1.54)**	1.79 (1.35–2.37)**
Readmission	1.83 (1.24–2.70)**	2.87 (1.58–5.2)**	1.71 (1.23–2.37)**	2.86 (1.88–4.35)**
Reoperation	0.75 (0.34–1.62)	1.90 (0.71–5.05)	1.52 (0.93–2.49)	1.18 (0.54–2.60)
NHD	1.07 (0.76–1.50)	0.80 (0.43–1.47)	1.79 (1.41–2.27)**	2.78 (1.83–4.21)**

*RAI* Risk Analysis Index, *mFI-5* 5-Item Modified Frailty Index, *NHD* Non-home discharge, *OR* Odds ratio, *CI* Confidence interval

^*^indicates significance < 0.05

^**^indicates significance < 0.01

**Fig. 1:**
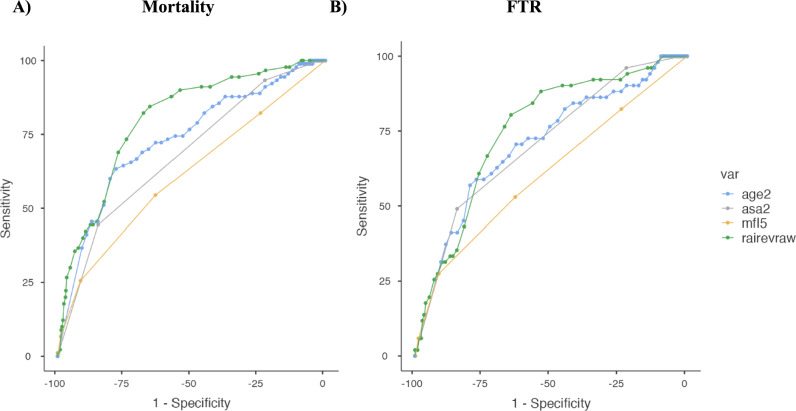
Receiver operating curves (ROC) demonstrating the discriminatory accuracy of the Risk Analysis Index (RAI), 5-item modified frailty index (mFI-5), Age, and American Society of Anesthesiologists (ASA) classification for predicting 30-day mortality (**A**) and failure to rescue (FTR) (**B**) after distal femur fracture (DFF) fixation. The area under the ROC (AUROC) curve, or C-statistic, quantifies the discriminatory accuracy with higher values indicating better ability to distinguish. RAI demonstrated highest discrimination for 30-day mortality and FTR. Blue curve predictive performance of Age, Grey curve predictive performance of ASA, Orange curve predictive performance of mFI-5, Green curve predictive performance of RAI

AUROC analysis was performed to assess whether RAI measure had a better discriminatory accuracy for determining postoperative outcomes (Table 3 and Fig. 1). Our analysis demonstrated that RAI (C-statistic = 0.80, 95% CI (0.75–0.85)) as a measure of frailty outperformed mFI-5 (C-statistic = 0.61, 95% CI (0.55–0.68)), age (C-statistic = 0.68, 95% CI (0.63–0.73)), and ASA classification (C-statistic = 0.73, 95% CI (0.68–0.78) in predicting mortality. Our analysis also showed that RAI (C-statistic = 0.75, 95% CI (0.68–0.83)) had an improved accuracy in predicting FTR compared to mFI-5 (C-statistic = 0.61, 95% CI (0.54–0.68)), age (C-statistic = 0.71, 95% CI (0.64–0.78)), and ASA classification (C-statistic = 0.71, 95% CI (0.64–0.78)).

**Table 3:** Results of AUROC analysis (DeLong test) demonstrating discriminatory accuracy of RAI, mFI-5, ASA Classification, and Age for predicting 30-day outcomes in distal femur fracture fixation

Outcome	RAI (C-statistic, 95% CI)	mFI-5 (C-statistic, 95% CI)	Age (C-statistic, 95% CI)	ASA (C-statistic, 95% CI)	p-value
Mortality	0.80 (0.75–0.85)**	0.61 (0.55–0.68)**	0.68 (0.63–0.73)**	0.73 (0.68–0.78)**	< 0.0001**
Failure to rescue	0.75(0.68–0.83)**	0.61 (0.54–0.68)**	0.71 (0.64–0.78)**	0.71 (0.64–0.78)**	0.0016**
Major complications	0.64 (0.59–0.69)*	0.60 (0.55–0.65)*	0.60 (0.55–0.65)*	0.68(0.63–0.73)*	0.01*
Minor complications	0.58 (0.54–0.62)	0.57 (0.53–0.61)	0.56 (0.52–0.60)	0.60 (0.56–0.64)	0.61
Readmission	0.61 (0.57–0.65)**	0.63 (0.59–0.67)**	0.63 (0.59–0.67)**	0.53 (0.49–0.57)**	< 0.0001**
Reoperation	0.53 (0.46–0.59)	0.56 (0.50–0.62)	0.60 (0.54–0.66)	0.55 (0.49–0.62)	0.235
NHD	0.76 (0.73–0.78)**	0.65 (0.63–0.67)**	0.67 (0.65–0.69)**	0.77 (0.75–0.79)**	< 0.0001**

*RAI* Risk Analysis Index, *mFI-5* 5-Item Modified Frailty Index, *ASA* American Society of Anesthesiologists, *NHD* Non-home discharge, *OR* Odds ratio, *CI* Confidence interval

^*^indicates significance < 0.05

^**^indicates significance < 0.01

### Secondary Outcomes

Multivariate regression analysis (Table 2) showed that RAI classification of patients as frail and very frail was predictive of greater odds of readmission, but classification as very frail also had greater odds of major complications compared to normal patients. Comparatively, mFI-5 classification of patients as frail and very frail had greater odds of major complication, minor complication, readmission, and NHD compared to normal patients.

## Discussion

In this large multicenter cross-sectional study of 2638 patients undergoing operative fixation for DFFs, we found that increasing frailty, as measured by the RAI was superior for prediction and risk discrimination of 30-day mortality and FTR when compared to the mFI-5, ASA, or age alone. To our knowledge, this is the first study validating the use of RAI over age, mFI-5 and ASA in patients undergoing DFF fixation to predict postoperative mortality and FTR. These findings suggest that frailty status plays a crucial role in influencing postoperative outcomes following DFF surgical fixation and that RAI may be a more effective tool for preoperative risk stratification in this patient population.

DFFs are complex injuries that represent a significant burden and are associated with high morbidity and mortality rates [1]. Because of this, it is critical to develop risk stratification tools for vulnerable populations. Preoperative frailty, as measured by mFI-5, has shown to be an independent risk factor for worsened postoperative outcomes in patients undergoing orthopedic trauma surgery [13]. More recently, the RAI emerged, providing a comprehensive measurement by incorporating five domains of frailty [14]. Using both the RAI and mFI-5, our findings demonstrated that increasing frailty was a significant predictor of mortality and FTR following DFF fixation. Notably, patients classified as "very frail" by RAI had six times more likely odds of mortality within 30 days and were more than twice as likely to experience FTR compared to non-frail patients. Similarly, very frail patients per mFI-5 had over threefold increased odds of mortality and FTR. Secondary outcomes further support the clinical relevance of frailty. Very frail patients, per both RAI and mFI-5, had significantly higher odds of hospital readmission and major complications. In addition, patients identified as frail or very frail by mFI-5 also had increased odds of minor complications and non-home discharge, indicating a greater need for postoperative care and rehabilitation.

Although previous literature measuring the predictive ability of frailty for postoperative outcomes in DFFs is sparse, our findings are consistent with prior studies related to other types of orthopedic trauma fractures [13, 15, 16]. Recent studies evaluating hip fracture populations have demonstrated similar results with the predictability of RAI. In a cross-sectional study with more than 100,000 patients with hip fractures, increasing frailty was significantly associated with greater odds of 30-day mortality [16]. Their results also showed that RAI had superior discrimination for 30-day mortality compared with mFI-5 [16]. This parallel between hip fractures and DFFs suggests that frailty is a useful tool in predicting postoperative mortality in orthopedic trauma patients, and that RAI may be a better indicator of physiologic reserve compared to other risk stratification tools in this population. Taken together, our findings underscore the potential utility of frailty assessment in the perioperative management of patients undergoing DFF fixation.

The significant association between frailty and mortality/FTR highlights an opportunity to use this metric as a risk stratification tool. RAI can be integrated into perioperative workflows as a frailty screening tool to guide tailored surgical decision-making, allocating perioperative resources adequately, and improving efficacy of postoperative monitoring. Patients with RAI scores greater than 30 are considered frail and may benefit from earlier engagement with geriatric or perioperative medicine teams to implement personalized care for each patient. By working with patients preoperatively, interprofessional teams can work to address modifiable risk factors, such as nutrition and prehabilitation, to better prepare their body for the stresses of surgery. Further, integration of RAI into electronic health records could help facilitate automatic flagging of high-risk patients and promote early multidisciplinary coordination, including physical therapy and enhanced ICU monitoring, to improve outcomes. RAI risk stratification may also guide discharge planning and transitional care services to ensure patients have appropriate support postoperatively. RAI is less of a binary decision-making tool, and provides more of a structured continuous risk stratification framework, allowing surgeons to make better perioperative decisions to improve outcomes in patients undergoing DFF fixation.

While both frailty indices were predictive of adverse outcomes, RAI consistently outperformed mFI-5 in terms of discrimination. ROC curve analysis showed that RAI had the highest C-statistic for predicting both mortality (0.80) and FTR (0.75), outperforming not only mFI-5 but also the widely used ASA classification. In contrast, the mFI-5 showed limited predictive accuracy, with C-statistics of only 0.61 for both outcomes. This aligns with prior literature that has suggested that RAI may be a more comprehensive frailty assessment tool compared to mFI-5 in predicting postoperative outcomes in patients across different surgical specialties [9, 17]. This can be attributed to the fact that RAI is a more comprehensive frailty assessment tool, as it includes more variables than the mFI-5. While mFI-5 primarily includes only comorbidity burden, RAI integrates many domains, including cognitive function, nutritional status, and social support, which provides a better understanding of the physiologic reserve and recovery likelihood of surgical patients. Outcomes, especially in orthopedic trauma, are strongly influenced by the factors included in RAI, providing a more holistic measure of patient vulnerability compared to mFI-5. Furthermore, the findings from this study supports RAI being a superior tool compared to ASA. ASA is widely used in preoperative risk assessment due to its widespread clinical applicability and familiarity. However, it is largely a subjective measure and lacks the multidimensional domains that RAI includes, which strongly influence a patient’s frailty status. Therefore, RAI, which captures key components that determine a patient’s baseline physiologic reserve, may be a better tool than ASA in predicting outcomes such as mortality and FTR, postoperatively.

Not only does the ROC curve analysis show that the RAI offers statistically superior discriminatory accuracy, but it also has demonstrated clinical relevance that may support meaningful changes in healthcare practice. This is exemplified by RAI’s integration into routine practice for preoperative frailty assessment. Its incorporation into widely used electronic health records like Epic and Cerner makes it accessible to clinicians in various settings [11]. Many major U.S. hospitals and VA systems have also already adopted its use [11]. Furthermore, a major strength of the RAI is its efficiency—it can be completed in under 30 seconds during regular clinic visits without disrupting workflow, which is especially beneficial in high-volume healthcare systems [11]. Additionally, the RAI is embedded in surgical quality databases like the Vascular Quality Initiative (VQI) and the American College of Surgeons National Surgical Quality Improvement Program (ACS NSQIP), supporting its retrospective use [11]. It has also been validated using ICD-10 codes (RAI-ICD), enabling large-scale retrospective frailty assessments and research [11]. For example, Dicpinigaitis et al. used RAI-ICD to show quantification of frailty across both operative and nonoperative patient populations [18]. This further suggests the broad clinical applicability of RAI across different clinical settings, and underscores its importance in perioperative risk assessment and stratification. This combination of accessibility, speed, and versatility makes the RAI a practical and impactful tool for improving patient care through better risk assessment and tailored interventions.

### Limitations

This study has several limitations. We used the ACS NSQIP administrative database for collection of data, introducing the possibility of clerical errors which could be a source of selection bias. This database does not allow stratification for open vs. closed fractures or low-energy vs. high-energy fractures, introducing the potential for confounding, as these factors can contribute to outcomes. The retrospective nature of the database, along with coding inaccuracies can add bias to the results. The database also only includes 30-day postoperative events, which makes it difficult to assess how increasing frailty is associated with long-term mortality or FTR. This limits the scope of the predictive power of the frailty scale. The database may also be limited by the completeness of complications reporting, such as superficial surgical site infections, minor complications, and cardiopulmonary events, which may lead to an underreporting of these events. This can lead to underreporting the complication rates in this study, which could bias FTR estimates lower than true value. The database also lacks certain details related to perioperative care, such as timing of recognition of complications or institutional protocols that may affect FTR rates. Additionally, this study looked at the outcomes of surgical treatment of DFFs overall, rather than specific surgical strategies. Future studies may seek to stratify based on different DFF surgical strategies to evaluate the use of frailty measures in predicting 30-day mortality and FTR. Lastly, the retrospective nature of this study also poses a limitation as confounding variables could be affecting outcomes. Future studies may demonstrate the effect of prehabilitation on reducing 30-day mortality and FTR in frail patients.

Given the aging population and increasing prevalence of fragility fractures, having accurate preoperative risk stratification is critical. Our findings suggest that frailty is a significant and independent predictor of mortality and FTR following surgical treatment of DFFs in elderly patients. Compared to mFI-5 and ASA classification, the RAI demonstrated superior predictive accuracy and may serve as a more effective tool for identifying high-risk patients. Orthopedic surgeons should consider adopting RAI in preoperative workflows to enhance risk stratification, optimize care pathways, and improve outcomes for this vulnerable patient population.

## Data Availability

Data can be made available upon request from authors.
